# Reply to: No evidence of worsening Arctic springtime ozone losses over the 21st century

**DOI:** 10.1038/s41467-023-37135-2

**Published:** 2023-03-24

**Authors:** Peter von der Gathen, Rigel Kivi, Ingo Wohltmann, Ross J. Salawitch, Markus Rex

**Affiliations:** 1 Alfred Wegener Institute, Helmholtz Centre for Polar and Marine Research, Potsdam, Germany; 2 Finnish Meteorological Institute, Space and Earth Observation Centre, Sodankylä, Finland; 3 Department of Atmospheric and Oceanic Science, Department of Chemistry and Biochemistry, and Earth System Science Interdisciplinary Center, University of Maryland, College Park, MD USA; 4 Universität Potsdam, Institut für Physik und Astronomie, Potsdam, Germany

**Keywords:** Atmospheric chemistry, Atmospheric dynamics

replying to L. M. Polvani et al. *Nature Communications* 10.1038/s41467-023-37134-3 (2023)

Long-term declines in the thickness of Earth’s protective ozone layer have been caused by the decomposition products of organic chlorine and bromine compounds such as chlorofluorocarbons and halons that have been phased out by the Montreal Protocol and its amendments and adjustments. The decline of these industrial compounds, termed ozone-depleting substances (ODSs), will result in the recovery of the thickness of the ozone layer, including eventually the elimination of severe loss of ozone in polar regions during this century^1^. We had written “future levels of Arctic column ozone during late winter and early spring are expected to increase due to factors such as intensification of the Brewer Dobson Circulation (BDC), upper stratospheric cooling, as well as possible changes in planetary and gravity wave activity that exert a strong influence on the abundance of column ozone within the Arctic vortex”^2^.

Polvani et al.^3^, hereafter P22, question our finding that conditions favorable for extensive chemical loss of ozone in the Arctic stratosphere during early spring could persist, or worsen, if future abundances of greenhouse gases (GHGs) continue to steeply rise, despite the expected future decline in the abundance of ODSs^2^. They suggest our study is flawed due to use of an empirical proxy, termed the ozone loss potential (OLP), to ascertain how climate change might affect steep reductions of column ozone in the Arctic stratosphere during exceptionally cold, future Arctic winters. Regions of steep reduction in total column ozone (TCO) are caused by chemical loss due to the decomposition products of ODSs^4–6^. The transformation of inorganic chlorine from benign to highly reactive forms by heterogeneous reactions that takes place on the surface of polar stratospheric clouds (PSCs), which form only during particularly cold winters, initiates the chemical loss of Arctic ozone^7–10^. The persistence into early spring of conditions cold enough to support the formation of PSCs is responsible for record levels of ozone loss that occurred in the Arctic during March of 2011 (ref. ^5^) and March 2020 (ref. ^6^).

P22 present projections of total column ozone for the month of March from five Coupled Model Intercomparison Project Phase 6 (CMIP6) models that include interactive chemistry. Their analysis and that of the literature they cite^11–16^ is based on column ozone averaged over 60° to 90°N latitude (hereafter “polar cap*”*) for the month of March, whereas our analysis focuses on the extent of chemical loss of column ozone in the dynamically distinct Arctic “polar vortex*”* ^17^ during specific winter/early-spring seasons prior to vortex breakup^5,6,18,19^. Figure 1 shows total column ozone in the Arctic region averaged over March for three years (top row) and on specific days that experienced significant chemical loss (bottom row). Any analysis limited to the polar cap averaged over all of March will inevitably combine chemical reductions of stratospheric ozone within the Arctic vortex with transport-related enhancements of column ozone that occur outside the vortex, and will also include air masses with enriched ozone transported poleward following the breakup of the Arctic polar vortex. For these reasons, the model results and much of the criticism of P22 is misdirected.

**Fig. 1:**
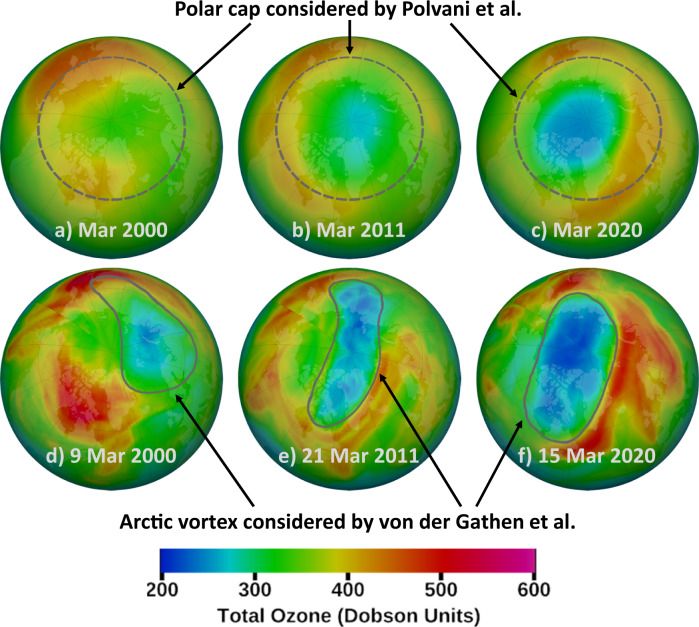
Total Column Ozone, Arctic, for selected years. The figures on the top (panels **a**–**c**) show the monthly mean total column ozone in DU for the Northern Hemisphere in March 2000, 2011, and 2020, respectively, as measured by the Total Ozone Mapping Spectrometer-Earth Probe (TOMS-EP) satellite (2000), the Ozone Monitoring Instrument (OMI) instrument onboard the Aura satellite (2011), and the Ozone Mapping and Profiler Suite (OMPS) instrument onboard the Suomi National Polar-orbiting Partnership (NPP) satellite (2020). Figures on the bottom (panels **d**–**f**) show the daily distribution of total column ozone on 9 March 2000, 21 March 2011, and 15 March 2020. Measurement gaps have been filled using data assimilation by the National Aeronautics and Space Administration (NASA) Goddard Space Flight Center Ozone Watch team, as described at https://ozonewatch.gsfc.nasa.gov. The gray dashed circle on panels **a**–**c** shows the polar cap (60° to 90°N) and the gray solid lines on panels **d**–**f** denote the Arctic polar vortex boundary found using the Nash^17^ criteria for the 475 K potential temperature level. The dates shown in panels **d**–**f** have been chosen to highlight the mismatch between the Arctic vortex analyzed in ref. ^2^ and the polar cap region favored in ref. ^3^. The low levels of column ozone within the Arctic vortex are largely caused by chemical loss due to reactions involving anthropogenic halogens; averaged over the entire month of March the region of depleted column ozone is considerably smaller than the polar cap, as shown for 2020 by Fig. 1c of Weber et al.^30^.

Even if we limit ourselves to the polar cap region for March, it is clear that many of the earlier models with interactive chemistry analyzed by Dhomse et al.^12^ and the CMIP6 models analyzed by P22 have difficulty representing observed TCO, ultimately due to deficiency in the model representation of chemical ozone loss. To achieve an accurate model representation of chemical ozone loss, models must accurately simulate temperature and humidity in the polar vortex, must have proper representation of the physical isolation of air within the vortex, and must also simulate well the descent of air and associated transport of chlorine species^20^. Figure 11 of ref. ^12^ shows stark differences between observed and simulated TCO in the Arctic polar cap for March, with models failing to capture steep lows observed during particularly cold winters. Much of the published analysis of model output is based on multi-model-means (MMM). Keeble et al.^11^ state “the CMIP6 MMM underestimates the observed decline in TCO for March in the NH polar regions during the ozone depletion period (1980–2000)”. This shortcoming is readily apparent in Fig. 6f of ref. ^11^. In contrast to ref. ^11^, P22 base their analysis on the subset of five CMIP6 models with interactive chemistry.

We analyzed output from these five CMIP6 models shown by P22 with respect to observed temperature in the Arctic lower stratosphere. The CESM2-WACCM, MRI-ESM2-0 and UKESM1-00-LL models have warm biases of 2 K, 2 K, and 1 K, respectively, whereas the CNRM-ESM2-1 has a substantial cold bias of about 4 K (ref. ^2^). The GFDL-ESM4 model^21^ has a warm bias of about 9 K. Most of the models highlighted by P22 likely underestimate the chemical loss of column ozone, since the proper simulation of the formation of PSCs and heterogeneous reactions that occur on PSCs requires reasonably accurate model representation of temperature. Three (GFDL-ESM4, MRI-ESM2-0, CESM-WACCM) of the five models shown in Fig. 1d of P22 show little to no reductions in column ozone during March for the decades when ODS abundances maximized. The GFDL-ESM4 CCM never achieves temperatures low enough to allow for the formation of PSCs, either historically or in the future for the SSP5-8.5 run with quite large radiative forcing (RF) of climate, despite future cooling of the Arctic vortex in the archived model output for this run. P22 state “large and sustained emissions of CO_2_ are not accompanied by large [future] ozone losses” based on analysis of output from these models. We are not surprised, since as shown in Fig. 1c of P22, three models (CESM2-WACCM, GFDL-ESM4 and MRI-ESM2-2) fail to properly simulate minimum values of TCO over 1979–2021, such as observed in 2011 and 2020. In the actual stratosphere, these winters experienced severe chemical loss of ozone by industrial halogens due to particularly cold conditions^5,6^. Given the fact these CCM runs are not constrained by observed meteorology, we would not expect minima TCO to be matched for the specific years during which these minima occur. However, an accurate simulation of the chemical loss of Arctic ozone should approach these TCO minima for the polar cap region for some winters. Another model (CNRM-ESM2-1) consistently underestimates the observed TCO minima in 2011 and 2022, perhaps due to the 4 K cold bias of Arctic stratosphere temperature apparent in our analysis of archived output from this model. Therefore, we suggest the statement by P22 that “Arctic ozone column in these models are consistent with the observed ozone column over the period 1979–2021” is misleading. Finally and most importantly, P22 fail to show results specific to the polar vortex, which is the well-established manner for examining chemical loss of ozone in the Arctic stratosphere^4–10^. By showing results only for the polar cap, the model projections of P22 are dominated by the expected future increase in TCO due to rising GHGs that occurs outside of the vortex.

The formation and existence of polar stratospheric clouds in the Arctic stratosphere is sensitive to ambient temperature as well as the abundance of H_2_O vapor. The abundance of H_2_O observed in the Arctic stratosphere is significantly underestimated by many CMIP6 models, in part because the source of stratospheric H_2_O from the oxidation of CH_4_ is either neglected or “under-represented” in many of these models^11^. This underestimation of stratospheric H_2_O, as large as a factor of two in some cases, will lead to an unrealistically low-temperature threshold for the formation of PSCs that further exacerbates the accurate representation of chemical ozone loss within these models. Consequently, we have used a well-established empirical approach to compute the production of stratospheric H_2_O from the oxidation of CH_4_ in our OLP proxy^2^. The suggestion of P22 that a more realistic depiction of future loss of Arctic ozone would result from the use of simulated values of H_2_O within the Arctic stratosphere seems odd in light of this recently published, quite dramatic model deficiency with respect to observed stratospheric H_2_O. Most importantly, even for the case of constant H_2_O we project in the supplement (Fig. S20)^2^ an OLP by the end of century that is about two-thirds that of the OLP found for the cases where H_2_O rises due to increases in CH_4_ and tropopause temperature.

P22 state “recent Arctic ozone minima are not related to increased level of CO_2_, but to the presence of ODS”. Here, we interpret their statement to mean that increased levels of CO_2_ do not amplify the impact of the ODSs on ozone, since we all agree ODSs are the cause of chemical loss of Arctic ozone. Confirmation^22^ of the predicted^23^ cooling of Earth’s stratosphere over the past half century due to rising levels of GHGs constitutes an important component of the overwhelming evidence that global warming is caused by humans. Rex et al.^18^ first suggested rising levels of GHGs cause winters in the Arctic stratosphere to become colder in a manner that favors the enhanced formation of PSCs. A strong correlation between the chemical loss of column ozone and various measures of PSC abundance within the Arctic vortex has been conclusively demonstrated^18,19,24–27^. Pommereau et al.^28^ also suggested rising levels of GHGs are responsible for cooling of the lower stratosphere in a manner that might significantly delay the projected recovery of Arctic ozone; however, P22 erroneously claim that this paper supports their view that Arctic ozone is insensitive to CO_2_. In ref. ^2^, we showed that a quantity termed PSC formation potential (PFP) within the Arctic vortex computed by CMIP5/6 models is projected to vary, in the coming decades, in a manner directly related to the RF of climate by GHGs. In our Methods section^2^, we provide a detailed description of the fallacy of Rieder and Polvani’s^29^ approach to assess the statistical significance the volume of air in the Arctic vortex exposed to PSCs, which was conducted prior to the record-breaking Arctic winter of 2020. Consequently, we dispute the contention by P22 that “recent Arctic ozone minima are not related to increased levels of CO_2_^”^.

The focus by P22 on the polar cap as well as their lack of critical appraisal of model behavior obscures the chemical loss of ozone within the Arctic vortex, which was the topic of our study^2^. We encourage the modeling community to devise metrics to improve the accuracy of the representation of chemical ozone loss of Arctic ozone within global models, a task that will require concerted efforts to assess modeled temperature, H_2_O, CH_4_, and simulated levels of inorganic halogens, as well as chlorine activation and the persistence of low temperatures into early spring, the sedimentation of PSCs, as well as transport of ozone by the BDC. If the rapid rise in the local maxima of PFP apparent in data from four meteorological centers over the past four decades is indeed being driven by the response of the climate system to rising levels of GHGs^2^, then the atmospheric sciences community would benefit from having computational tools that can be used to reliably evaluate the resulting chemical loss of Arctic ozone as well as the impact on total column ozone. Contrary to the view of P22, we suggest our OLP constitutes an important empirically based metric^2^ for evaluating the quantitative representation of chemical loss of column ozone within these global models. We conclude by noting that even though P22 have chosen to use the word “alarmist” to characterize the message of our original paper, the central message of this paper^2^ (i.e., further increases in the abundance of GHGs will result in conditions conducive to extensive chemical loss of stratospheric ozone by anthropogenic halogens in the Arctic polar vortex) is based upon analysis of a wide variety of measurements from a multitude of orbital and sub-orbital observing platforms that have benefited from an extraordinary amount of peer-review over the past quarter-century^4–10,24–28^.

## Data Availability

GFDL-ESM4 model outputs are provided by the World Climate Research Programme’s Working Group on Coupled Modelling and are available at https://esgf-node.llnl.gov/search/cmip6. Other model data are included in ref. ^2^.

## References

[1] Salawitch, R. J. et al. *Twenty Questions and Answers About the Ozone Layer: 2018 Update, Scientific Assessment of Ozone Depletion: 2018, 84 pp.* (World Meteorological Organization, Geneva, Switzerland 2019).

[2] von der Gathen P, Kivi R, Wohltmann I, Salawitch RJ, Rex M (2021). Climate change favours large seasonal loss of Arctic ozone. Nat. Commun..

[3] Polvani, L. M. et al. No evidence of worsening Arctic springtime ozone losses over the 21st century. *Nat. Commun.*10.1038/s41467-023-37134-3 (2023). 10.1038/s41467-023-37134-3 PMC10039004 36964124

[4] Manney GL (2020). Record-low Arctic stratospheric ozone in 2020: MLS observations of chemical processes and comparisons with previous extreme winters. Geophys. Res. Lett..

[5] Manney GL (2011). Unprecedented Arctic ozone loss in 2011. Nature.

[6] Wohltmann I (2020). Near-complete local reduction of Arctic stratospheric ozone by severe chemical loss in spring 2020. Geophys. Res. Lett..

[7] Brune WH (1991). The potential for ozone depletion in the Arctic polar stratosphere. Science.

[8] Salawitch RJ (1993). Chemical loss of ozone in the Arctic polar vortex in the winter of 1991–1992. Science.

[9] Waters JW (1993). Stratospheric ClO and ozone from the Microwave Limb Sounder on the Upper Atmosphere Research Satellite. Nature.

[10] Harris NRP, Lehmann R, Rex M, von der Gathen P (2010). A closer look at Arctic ozone loss and polar stratospheric clouds. Atmos. Chem. Phys..

[11] Keeble J (2021). Evaluating stratospheric ozone and water vapour changes in CMIP6 models from 1850 to 2100. Atmos. Chem. Phys..

[12] Dhomse SS (2018). Estimates of ozone return dates from Chemistry-Climate Model Initiative simulations. Atmos. Chem. Phys..

[13] Eyring V (2013). Long-term ozone changes and associated climate impacts in CMIP5 simulations. J. Geophys. Res. Atmos..

[14] Eyring V (2010). Multi-model assessment of stratospheric ozone return dates and ozone recovery in CCMVal-2 models. Atmos. Chem. Phys..

[15] Eyring, V. et al. Multimodel projections of stratospheric ozone in the 21st century. *J. Geophys. Res. Atmos*. **112**, D16303 (2007).

[16] Eyring, V. et al. Sensitivity of 21st century stratospheric ozone to greenhouse gas scenarios. *Geophys. Res. Lett*. **37**, L16807 (2010).

[17] Nash ER, Newman PA, Rosenfield JE, Schoeberl MR (1996). An objective determination of the polar vortex using Ertel’s potential vorticity. J. Geophys. Res. Atmos..

[18] Rex, M. et al. Arctic ozone loss and climate change. *Geophys. Res. Lett*. **31**, L04116 (2004).

[19] Rex, M. et al. Arctic winter 2005: implications for stratospheric ozone loss and climate change. *Geophys. Res. Lett*. **33**, L23808 (2006).

[20] SPARC CCMVal. *SPARC Report on the Evaluation of Chemistry-Climate Models*. SPARC Report No. 5, WCRP-132, WMO/TD-No. 1526 (eds Eyring, V. et al.) (World Meteorological Organization, 2010).

[21] Krasting, J. P. et al. NOAA-GFDL GFDL-ESM4 model output prepared for CMIP6 CMIP. Version 20220811. *Earth Syst. Grid Federation*. 10.22033/ESGF/CMIP6.1407 (2018).

[22] Santer BD (2013). Human and natural influences on the changing thermal structure of the atmosphere. Proc. Natl Acad. Sci. USA.

[23] Boughner, R. E. The effect of increased carbon dioxide concentrations on stratospheric ozone. *J. Geophys. Res*. **83**, 1326–1332 (1978).

[24] Tilmes, S., Müller, R., Engel, A., Rex, M. & Russell III, J. M. Chemical ozone loss in the Arctic and Antarctic stratosphere between 1992 and 2005. *Geophys. Res. Lett*. **33**, L20812 (2006).

[25] Tilmes S, Müller R, Grooß J-U, Russell JM (2004). Ozone loss and chlorine activation in the Arctic winters 1991–2003 derived with the tracer-tracer correlations. Atmos. Chem. Phys..

[26] Chipperfield, M. P., Feng, W. & Rex, M. Arctic ozone loss and climate sensitivity: updated three-dimensional model study. *Geophys. Res. Lett*. **32**, L11813 (2005).

[27] Tilmes, S. et al. Evaluation of heterogeneous processes in the polar lower stratosphere in the Whole Atmosphere Community Climate Model. *J. Geophys. Res. Atmos*. **112**, D24301 (2007).

[28] Pommereau J-P (2018). Recent Arctic ozone depletion: is there an impact of climate change?. Comptes Rendus Geosci..

[29] Rieder HE, Polvani LM (2013). Are recent Arctic ozone losses caused by increasing greenhouse gases?. Geophys. Res. Lett..

[30] Weber M (2021). The unusual stratospheric Arctic winter 2019/20: chemical ozone loss from satellite observations and TOMCAT chemical transport model. J. Geophys. Res. Atmos..

